# Ribosome profiling: a Hi-Def monitor for protein synthesis at the genome-wide scale

**DOI:** 10.1002/wrna.1172

**Published:** 2013-05-20

**Authors:** Audrey M Michel, Pavel V Baranov

**Affiliations:** Biochemistry Department, University College CorkCork, Ireland

## Abstract

Ribosome profiling or ribo-seq is a new technique that provides genome-wide information on protein synthesis (GWIPS) *in vivo*. It is based on the deep sequencing of ribosome protected mRNA fragments allowing the measurement of ribosome density along all RNA molecules present in the cell. At the same time, the high resolution of this technique allows detailed analysis of ribosome density on individual RNAs. Since its invention, the ribosome profiling technique has been utilized in a range of studies in both prokaryotic and eukaryotic organisms. Several studies have adapted and refined the original ribosome profiling protocol for studying specific aspects of translation. Ribosome profiling of initiating ribosomes has been used to map sites of translation initiation. These studies revealed the surprisingly complex organization of translation initiation sites in eukaryotes. Multiple initiation sites are responsible for the generation of N-terminally extended and truncated isoforms of known proteins as well as for the translation of numerous open reading frames (ORFs), upstream of protein coding ORFs. Ribosome profiling of elongating ribosomes has been used for measuring differential gene expression at the level of translation, the identification of novel protein coding genes and ribosome pausing. It has also provided data for developing quantitative models of translation. Although only a dozen or so ribosome profiling datasets have been published so far, they have already dramatically changed our understanding of translational control and have led to new hypotheses regarding the origin of protein coding genes. © 2013 John Wiley & Sons, Ltd.

## INTRODUCTION

The race for the completion of the human genome yielded a by-product that is probably more important for modern biology than the goal of the project itself—cheap and powerful technologies for sequencing DNA. These technologies shifted the focus of researchers from studying individual molecules and pathways to studying the whole composition of molecules inside the cell. However, most of the popular high-throughput techniques provide only static information on the composition of the cell. For example, proteomics approaches such as mass-spectrometry give information on the composition of a proteome, while RNA-seq captures information on the composition of a transcriptome. An assumption is used whereby the abundance of transcripts can be interpreted as a measure of transcription levels. This assumption is problematic because of the varying stability of RNA transcripts. Because of the high variability in protein molecule half-lives, inferring gene expression levels from protein abundance is even more problematic. A high concentration of a particular protein in the cell does not necessarily mean that the corresponding gene is being highly expressed at the moment of measurement.

**FIGURE 1:**
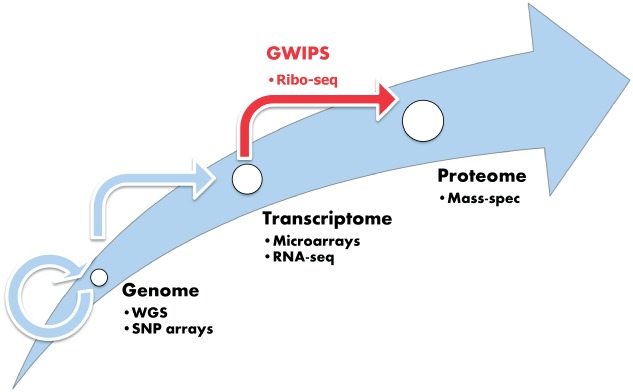
The emplacement of genome-wide information on protein synthesis (GWIPS) and the role of ribo-seq in characterizing the molecular status of the cell.

Until recently, no simple high-throughput technique existed for measuring gene expression at the level of translation. The situation has changed with the advent of the ribosome profiling technique developed in the laboratory of Jonathan Weissman at University of California, San Francisco.^1^ By providing genome-wide information on protein synthesis (GWIPS), ribosome profiling filled the technological gap existing between our abilities to quantify the transcriptome and the proteome^2^ (see Figure 1). It is now possible not only to detect RNA and protein molecules in the cell, but also determine which protein molecules are being synthesized in the cell at any given moment and therefore quantitatively measure the immediate reaction of the cell to a change in its internal environment.

**FIGURE 2:**
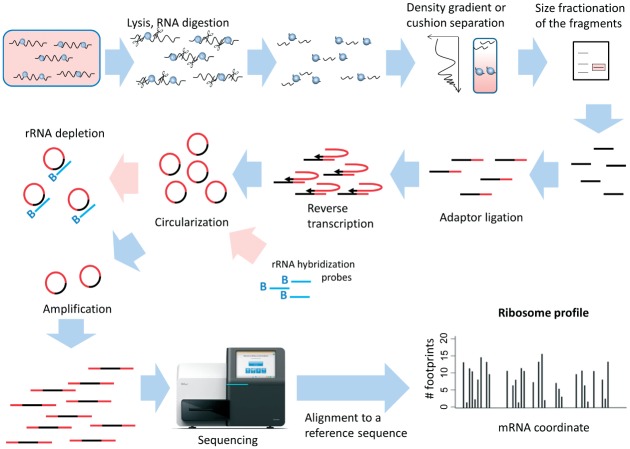
Outline of the major steps of the ribosome profiling protocol as described in Ingolia et al.^4^ The experimental part of the protocol requires 7 days. Modifications of the protocol have been made in several other studies and commercial kits for ribosome profiling are currently available.

The technology is the product of a propitious marriage of an existing methodology with massive parallelization offered by second-generation sequencing platforms.^1^ The ability of ribosomes to protect mRNA fragments from nuclease digestion has been used since the 1960s.^3^ In ribosome profiling (see Figure 2), this procedure is carried out for the entire cell lysate generating a pool of ribosome protected fragments or footprints (RPFs). Recovered footprints are converted to a format suitable for massively parallel sequencing. Analysis of the resultant sequences allows the quantification of ribosomes translating mRNAs at a genome-wide scale.^1^,^4^,^5^ Therefore ribosome profiling can be used for measuring gene expression at the translational level. However, this was already possible with polysome profiling where a pool of translated mRNAs is isolated from the polysome fraction of a sucrose gradient. This approach, where the abundance of transcripts in a polysome fraction is assessed either with RNA-seq or microarray techniques, has become a popular way of identifying genes whose expression is under translational control.^6^–^9^ The real power of ribosome profiling in comparison with such approaches is in its ability to obtain position-specific information regarding ribosome locations on mRNAs. This is very important for several reasons. The association of an mRNA transcript with ribosomes does not necessarily mean that the main open reading frame (ORF) of this mRNA is translated. Ribosomes could stall on an mRNA transcript without producing a protein. Translation could occur at ORFs other than the main protein coding open reading frame (pORF).

**FIGURE 3:**
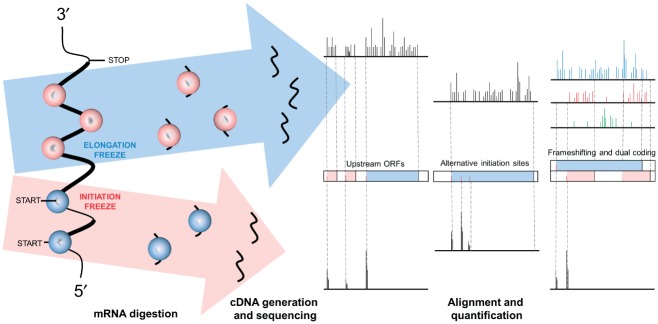
Two main ribo-seq strategies: ribosome profiling of elongating ribosomes (top, blue arrow) and ribosome profiling of initiating ribosomes (bottom, light-pink arrow). In both cases, the freezing of ribosomes at specific stages of translation is followed by the degradation of mRNA unprotected by ribosomes and subsequent preparation of ribosome footprint cDNA libraries and their sequencing. The right-hand side of the figure illustrates how the data obtained with these ribo-seq techniques can be analyzed for the identification of uORFs (shown as pink areas in the left plot), protein isoforms with alternative N-termini (middle plot), and nORFs embedded within annotated coding regions and recoding events (far-right plot).

Because ribosome profiling reveals the exact positions of ribosomes on an mRNA transcript, two major variants of the technique have been developed: ribosome profiling of elongating ribosomes and ribosome profiling of initiating ribosomes. Elongating ribosomes can be blocked with antibiotics that inhibit either translocation (e.g., cyclohexamide^1^ and emetine^10^), peptidyl transfer (e.g., chloramphenicol), or by thermal freezing.^11^ Information on the positions of initiating ribosomes can be obtained either by the direct blocking of initiating ribosomes with specific drugs (e.g., harringtonine^10^ and lactimidomycin^12^) or by enriching elongating ribosomes near the starts by blocking them with cyclohexamide following pretreatment with puromycin that causes premature termination.^13^ Figure 3 illustrates how these two distinct strategies can be used for the characterization of different phenomena. For certain applications each approach has its own advantages, for example, information on initiating ribosomes cannot be used for the detection of ribosomal frameshifting, while the detection of internal sites of initiation is impractical without this information. Often, these approaches complement each other and can be very powerful if used in parallel as has been demonstrated in a recent study.^14^ For clarity, and to emphasize the advantages of each strategy, this review is split into two main sections addressing each strategy separately.

## RIBOSOME PROFILING OF ELONGATING RIBOSOMES

The objective of using ribosome profiling is to generate a snapshot of the mRNAs that are being translated, capturing the exact locations of translating ribosomes and their densities on these mRNAs. It is imperative that the RPFs recovered from cell extracts accurately reflect the *in vivo* status of translation at the time of the experiment. Depending on the organism, the tissue and the objective of the study, the cell lysate preparation will vary. To faithfully capture elongating ribosomes in their *in vivo* translational positions, the majority of ribo-seq experiments to date have treated cells with translation elongation inhibitors to immobilize polysomes prior to cell lysis, followed by nuclease digestion. The nuclease-resistant RPFs are then recovered, converted to cDNA libraries and sequenced using massively parallel platforms (see Figures 2 and 3).

The elongation inhibitor cycloheximide^15^ has been used in nearly all of the elongating ribosome profiling studies carried out in eukaryotic cells to date. However, simple liquid nitrogen freezing as well as other antibiotics such as emetine in eukaryotes and chloramphenicol in bacteria have also been used.^1^,^10^,^11^ It is likely that the repertoire of translation inhibitors used in ribosome profiling studies will grow in the future, such as drugs that interfere with translation by stabilizing particular ribosomal conformations and thereby provide advantages for specific applications. It has been observed, for example, that the length of RPFs could be drug dependent.^10^

For details of the ribosome profiling experimental protocol see Refs 4,5 as well as the methods section of the primary research articles described in this review. In this section we will review the various applications of ribosome profiling of elongating ribosomes such as measuring differential gene expression, estimating global and local translation elongation rates and the identification of novel genes and the products of their expression.

### Differential Gene Expression Using Ribosome Profiling

The ability to detect changes in the expression of genes is essential for understanding the genetic determinants of phenotypical behavior and the molecular response of the cell to changing conditions. For more than a decade, microarray techniques,^16^ and more recently RNA-seq,^17^ have been used for measuring differential gene expression. However, the correlation between mRNA abundance and protein levels is insufficient for predicting protein expression based on mRNA concentrations (for discussion see Refs 18,19). Measurements of global protein and mRNA compositions have demonstrated that an important factor determining the cellular protein abundance in mammalian cells is its rate of translation.^20^ As discussed in the *Introduction* section, to obtain information on translated mRNAs, microarray and RNA-seq techniques can be applied to quantify the mRNAs bound to ribosomes by isolating the mRNAs from the polysome fractions of sucrose gradients. However, such a methodology is inaccurate. Two mRNA molecules in a polysome fraction could be translated at different rates, or not translated at all. This could occur, for example, when a ribosome is stalled on an mRNA or translation is limited to upstream open reading frames (uORFs) that often prevent translation of the main protein product ORF (removal of the monosomal fraction solves this issue for mRNAs inhibited with a single short uORF). Polysomal profiling also cannot provide information on the exact number of ribosomes on mRNAs. Because ribo-seq allows localization of the ribosomes, this information can be assessed, therefore making it a preferential approach for differential gene expression. The very first ribosome profiling study showed a 100-fold range difference in the density of ribosome footprints across different yeast transcripts expressed at a relatively high level.^1^ The high variation in ribosome densities and the ability of ribo-seq to detect the variation, demonstrate the advantages of ribo-seq in comparison with prior approaches.

Most of the published ribosome profiling studies borrowed computational approaches from RNA-seq analysis for measuring differential gene expression levels. For a number of reasons, specifically discussed at the end of this subsection and illustrated with examples throughout the entire review, treating the density of ribosome footprints on an mRNA transcript as a direct measure of its translation may generate a number of artifacts. It is likely that specialized tools for the analysis of gene expression using ribo-seq will be developed in the future. In the meantime, however, adapting RNA-seq computational approaches is sensible for obtaining approximate information. Indeed, by using such approaches, a small number of ribosome profiling studies have already provided significant insights into certain important aspects of translational control.

#### The Effects of Stress Conditions on Translation

**FIGURE 4:**
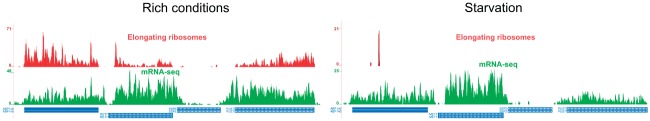
Ribo-seq (red) and mRNA-seq (green) coverage plots for the *S. cerevisiae* genome locus containing *ABP140*, *MET7*, *SSP2*, and *PUS7* genes obtained with GWIPS-viz (http://gwips.ucc.ie/) using data from Ref 1. Under starvation conditions (right), *ABP140*, *MET7* and *PUS7* are transcribed, but not translated.

Protein synthesis is an energetically expensive anabolic process and therefore it is expected to be sensitive to the available nutrition, in particular, amino acids. To test the ability of ribo-seq to characterize changes in protein synthesis in response to starvation, Ingolia et al.^1^ carried out ribosome profiling on yeast cells after 20 min of amino acid deprivation. Changes at the translational level were detected in approximately one-third of the 3769 genes that had sufficient coverage (see examples in Figure 4). For 291 genes, up- or downregulation was found to be greater than twofold. In particular, the translation of *GCN4* was found to increase sevenfold. While the translational regulation of *GCN4* in response to amino acid deficiency is well established and studied,^21^ this effect was not observed with a previous polysome profiling study.^22^ This example illustrates the clear advantage of ribosome profiling over polysome profiling as it allows the discrimination of mRNAs with efficiently translated coding regions from mRNAs where only the 5′UTRs are translated.

Geraschenko et al.^23^ used a similar idea to explore the translational response of *Saccharomyces cerevisiae* to oxidative stress. Yeast cells were treated with hydrogen peroxide and ribo-seq, and RNA-seq were carried out in parallel 5 and 30 min after the treatment. Many genes whose expression was altered at the transcriptional and translational level have been identified with this approach. The number of genes whose expression was changed greatly increased with the prolonged treatment: the transcript abundance of 116 genes was affected after 5 min and 1497 genes after 30 min with similar numbers obtained for genes whose translation was altered. Interestingly, they reported several transcribed but translationally quiescent genes whose translation is activated upon oxidative stress, for example, the *Srx1* gene which encodes sulfiredoxin. The dataset of translationally regulated genes was compared with a previous study that used polysome profiling for this purpose.^24^ About 70% of translationally regulated genes found with polysome profiling were not confirmed with ribosome profiling. Geraschenko et al.^23^ argue that such a large discrepancy could be due to the inability of polysome profiling to discriminate the translation of main ORFs from regulatory uORFs.

While this review was in preparation, two more studies were published that explored translational response to heat shock^25^ and to proteotoxic stress.^26^ Shalgi et al.^25^ found that 2 h of severe heat stress caused an accumulation of ribosomes in the first ∼200 nt of ORFs in mouse and human cells. Liu et al.^26^ found that proteotoxic stress in HEK293 cells resulted in elongation pausing primarily near the site where nascent peptides emerge from the ribosomal exit tunnel. Both studies discuss the role played by chaperones in translation elongation and that early elongation pausing is triggered when chaperones are sequestered to the misfolded protein response as a result of cellular stress.

#### The Role of miRNAs in Translational Control

The discovery of RNA interference (RNAi) opened up a debate regarding the potential mechanisms of translational regulation with miRNAs (see Refs 27–29). While many examples of RNAi inhibition of protein synthesis have been reported as well as cases of translational upregulation,^30^ the global contribution of RNAi to translational control is unclear. To address this issue, Guo et al. employed ribosome profiling in conjunction with mRNA-seq (alkaline-degraded mRNA yielding fragments of a size similar to ribosome footprints) to discriminate between changes in mRNA abundance and rates of protein production caused by the expression of specific miRNAs.^31^ The experiments were carried out in human HeLa cells using exogenous miRNAs.^31^ Genes with at least one miRNA target site in their 3′ UTRs were repressed by the addition of the corresponding miRNA resulting in fewer mRNA-seq fragments and correspondingly fewer RPFs. A very modest decrease in translational efficiency was observed for messages with miRNA target sites compared to those without. Therefore, Guo et al. concluded that, at the global level, miRNA interference affects mostly mRNA abundance with only a marginal effect on translation.^31^

However, as discussed by Janas and Novina,^32^ this study assessed translation and mRNA levels after 12–32 h, at which point only the downstream effects of miRNA function may have been observed. To study gene expression responses at earlier time points, Bazzini et al. carried out combined ribo-seq and mRNA-Seq analysis to study the global effects of a particular miRNA in zebrafish.^33^ For this purpose they focused on targets of miR-430 miRNA which is expressed at the onset of zygotic transcription and had been previously shown to promote deadenylation and degradation of maternal transcripts at 5 and 9 h postfertilization (hpf).^34^ The ribosome occupancy and mRNA levels of miR-430-targeted mRNAs were measured at timepoints before (2 hpf) and after (4 hpf and 6 hpf) the induction of miR-430 expression. At 4 hpf the ribosome density along miR-430-targeted mRNAs was uniformly decreased without a corresponding decrease in the mRNA. Yet 70% of the targets translationally repressed at 4 hpf were deadenylated or degraded at 6 hpf, suggesting that mRNA decay followed translational repression.

Stadler et al. performed parallel mRNA-seq and ribo-seq to analyze the translational changes in a set of five genes (lin-14, lin-28, daf-12, hbl-1, and lin-41) which are known targets of specific miRNAs during the different stages of larval development in *Caenorhabditis elegans*.^35^ The analysis of the obtained data suggested that miRNAs interfere with gene expression by mRNA destabilization, translation initiation inhibition, and probably through other translational events during elongation.

While these studies did not end the debate regarding the role and the mechanisms of miRNA-mediated translational control,^36^,^37^ they provided interesting insights into the process and demonstrate that the parallel application of ribo-seq and mRNA-seq is a powerful approach for delineating the transcriptional and translational controls of gene expression.

#### Characterization of the Role of Protein Regulators of Translation

mTOR is a kinase that regulates global protein synthesis by phosphorylating the protein 4E-BP whose unphosphorylated form inactivates initiation factor eIF4E whose function is to bind to the mRNA 5′-cap and initiate the assembly of the initiator ribosome complex.^38^ The mTOR pathway is dysregulated in many diseases particularly in cancer, where its dysregulation is manifested by uncontrollable cell growth and overactive protein synthesis.^39^,^40^ A number of genes directly regulating the mTOR pathway are well known tumor suppressors and oncogenes and it is not surprising that mTOR inhibitors emerged as potential agents for cancer therapy.^41^

Two recent works employed ribo-seq to study the translational regulation mediated by mTOR. Thoreen et al.^42^ carried out comparative ribo-seq analysis in mouse embryonic fibroblasts (MEFs). Treatment of MEFs with a potent mTOR inhibitor, Torin 1, resulted in the translational suppression of nearly all (99.8%) mRNAs, confirming mTOR's role as a global regulator of proteins synthesis. Hsieh et al.^43^ carried out ribosome profiling in PC3 human prostate cancer cells, where mTOR is constitutively hyperactivated, to capture changes in gene expression in response to treatment with another mTOR inhibitor, PP242. In addition to observing a global effect on translation, both studies explored a pool of mRNAs whose translation is particularly sensitive to mTOR inhibition. A 5′-terminal oligopyrimidine tract (TOP) is a common feature of genes that are translationally regulated in a growth-dependent manner.^44^,^45^ Hsieh et al.^43^ reported that 68% of mTOR sensitive mRNAs possess the TOP motif and 63% of such mRNAs contain a pyrimidine-rich translational element (PRTE) elsewhere within their 5′-UTRs. Overall 89% of mTOR sensitive mRNAs were found to contain either one or both motifs. Thoreen et al.^42^ were able to identify TOP or TOP-like motifs in almost the entire set of mTOR sensitive mRNAs. Therefore the presence of pyrimidine-rich sequences in 5′-UTRs can be used as a strong predictor of mRNA sensitivity to mTOR inhibition. These two studies illustrate the power of ribo-seq in helping researchers to characterize cellular signaling pathways whose dysregulation is implicated in human diseases such as cancer.^46^

In a recent work focused on the characterization of the RNA-binding protein LIN28A, Cho et al.^47^ used ribosomal profiling to assess LIN28A's role as a global regulator of translation. For this purpose, ribosome profiling was carried out in mouse embryonic stem cells (mESCs) after LIN28A knockdown. The knockdown resulted in an increased density of ribosomes on ER-associated mRNAs without affecting their levels. Based on these data, Cho et al. proposed that LIN28A is a major inhibitor of translation in the endoplasmic reticulum of undifferentiated cells.^47^

#### Temporal Translational Control

**FIGURE 5:**
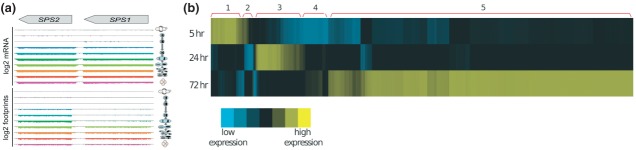
Examples of temporal translational control. Panel (a) shows the expression levels of the adjacent *SPS1* and *SPS2* genes at different stages of meiosis in *S. cerevisiae*. The mRNA levels are consistent throughout all stages of meiosis. However, the ribosome profiling data for *SPS1* shows strong temporal translational regulation while *SPS2* does not (Reprinted with permission from Ref 48. Copyright 2012 AAAS). Panel (b) provides a heatmap of the ribosome density of viral genes clustered according to expression levels at 5, 24, and 72 h after the infection of human foreskin fibroblasts with cytomegalovirus (Reprinted with permission from Ref 14. Copyright 2012 AAAS)

Brar et al. explored temporal changes in gene expression during meiosis in *S. cerevisiae*.^48^ Over stage-specific timepoints, ribosome profiling captured many dynamic events that occur during the progression of meiosis that were not detected with previous technologies. They found at least 10-fold variations in expression for 66% of genes. While most of these variations occur due to changes in the abundance of gene transcripts, ribo-seq also revealed pervasive translational regulation. At the global level, translation was decreased during meiosis, especially at its earliest and latest stages. Brar et al. also observed stage-specific regulation in the translation of individual mRNAs matching the timing of their products known function.^48^ Figure 5a provides an example of stage-specific translational regulation observed for the adjacent *SPS1* and *SPS2* genes. The mRNA levels for both genes showed comparable changes throughout the different stages of meiosis. Yet *SPS1*, but not *SPS2*, showed a strong temporal delay in the activation of its translation.

At the time of writing this review, Stern-Ginossar et al.^14^ published a study where temporal gene expression changes were analyzed during the infection of human foreskin fibroblasts with cytomegalovirus. Measurements were made 5, 24, and 72 h after infection. A strong temporal regulation of viral gene translation was observed with the translation of 82% of ORFs varying at least fivefold.^14^ Figure 5b shows a heatmap of viral ORF translation levels illustrating the temporal control of protein synthesis. Different groups of ORFs are translated at different time points with the majority switched on at the last stage.

#### The Need for Specialized Computational Tools for Differential Expression Analysis Using ribo-seq and *RNA-seq Data*

Obviously two transcripts expressed at the same level but of different length would produce a different number of short reads aligning to them as the number of reads is proportional to the length of the transcript. Thus the absolute number of short reads derived from a particular transcript is usually normalized to the length of the transcript as well as to the total number of alignable reads, as in Cufdiff FKPM units.^49^ Similarly, the transcript length needs to be taken into account when measuring the relative translation of two mRNAs because the time that ribosomes would spend on the mRNAs would differ depending on the length of the translated ORF.^5^ Because ribosomes broadly translate mRNAs at a similar elongation rate,^10^ conversion of the absolute number of footprints into ribosome density can be used for estimating translation rates. However, this is likely to be useful only as a broad approximation because of the high variance in the time that ribosomes decode individual codons, for example, sequence- and condition-dependent pausing and stalling, and also because of the complex organization of eukaryotic mRNA translation at 5′ UTRs. Clearly an mRNA containing paused ribosomes is not translated as efficiently as an mRNA that is covered with fast paced ribosomes even though the density of ribosomes could be similar for both of them.

The notion that only a single ORF is translated in an individual eukaryotic mRNAs and that 5′-UTR stands for ‘untranslated’ terminal region are mostly of historical interest after the discovery of functional regulatory uORFs.^50^ The term *5*′ *leader* seems to be an adequate substitute to avoid the oxymoron ‘translation of 5′-UTRs’. The frequent occurrence of conserved AUGs in 5′ leaders was revealed by phylogenetic analyses.^51^ The extensive translation of 5′ leaders has been well supported by ribosome profiling studies described in this review. This implies that the ribosome density and the efficiency of the mRNA main protein product synthesis may not correlate perfectly. The ribosome footprints that originate from uORFs contribute to the overall footprint coverage of a given mRNA transcript and can affect the correct quantification of the ribosome density in a pORF. At a minimum it necessitates the discrimination of ribosome density in the 5′ leaders from CDS regions when quantifying RPFs for protein synthesis measurements. While such discrimination would improve the assessment of the rate of main protein product ORF translation, it is unlikely to be applicable to all mRNAs because of the existence of uORFs overlapping the main ORF and also the existence of nonupstream or nested ORFs (nORFs) contained within main ORFs discovered with the analysis of published ribo-seq data.^52^ In this case, footprints aligning to the pORF do not necessarily indicate its translation. Separating footprints originating from overlapping uORFs and nORFs from footprints originating from annotated pORFs can be problematic. The use of the triplet periodicity property of ribosome profiling and the generation of subcodon profiles^52^ can help to solve this conundrum. If the ribo-seq data has well-defined triplet periodicity such as in the Guo et al. study,^31^ the footprints originating from ORFs in frames alternative to the pORF can be detected, thus permitting the correct quantification of pORF translation levels.

Another problem related to differential translation measurement lies in the method for normalizing translation efficiency over mRNA abundance. A change in mRNA abundance due to changes in transcription or mRNA stability would ultimately result in a corresponding change in the number of ribosome footprints. A simple approach to take this into account is to compare log ratios of ribosome densities over mRNA abundance. Hence, mRNA-seq data, generated in parallel with ribo-seq data, is used to correct for a possible contribution of differential cytosolic mRNA levels to the observed differential levels of actively translated mRNAs. However, Larsson et al. caution against using the commonly applied log ratio approach (ribo-seq levels divided by corresponding mRNA-seq levels) because log difference scores could correlate with cytosolic mRNA levels. The possible confounding effect of cytosolic mRNA levels may result in biological false positives and false negatives. As an alternative, Larsson et al. proposed analysis of partial variance (APV) as a more accurate correction method for cytosolic mRNA levels.^7^ Their implementation is available in the R-package anota (analysis of translational activity) for the analysis of differential translation using ribosome profiling datasets as well as polysome microarray or RNA-seq-based datasets.^53^

A limitation of ribosome profiling is that it allows to measure only relative changes in gene expression. Because ribo-seq does not provide information on absolute changes of translation, global suppression of translation may be misinterpreted as the activation of translation of a few unaffected genes. In RNA-seq experiments, this problem is solved with the addition of synthetic RNA molecules with a different nucleotide composition (spike-in control).^54^ Han et al.^55^ adapted this idea by adding a synthetic 28-nt long oligonucleotide that mimics the ribosome footprint. It is desirable that standard spike-in controls will be developed and accepted by the community to allow for comparison of datasets between labs.

### Estimating Global Average and Local Rates of Translation Elongation

Prior to ribosome profiling, measurements of translation elongation rates were carried out on individual mRNAs.^56^,^57^ To estimate the global average rate of translation elongation, Ingolia et al. used a pulse-chase strategy by preventing new translation initiation using harringtonine followed by a short time for run-off elongation before adding cycloheximide.^10^ The experiments carried out in mESCs demonstrated that ribosomes progress on mRNA transcripts at an average rate of ∼5.6 codons per second.^10^ The rate of elongation is consistent across different types of mRNAs, independent of the length and abundance of encoded proteins. It is also uniform across the length of the coding region beyond the initial 5–10 codons. By analyzing the same data using a different approach, Dana and Tuller^58^ concluded that while the average translation velocity of all genes is ∼5.6 amino acids per second, the speed of elongation is slower at the beginning of coding regions and linked this observation to a decrease in the strength of the mRNA folding along the coding sequence and a decreased frequency of optimal codons in these regions, known as the ‘ramp theory’.^59^

The common interpretation of ribosome profiling data is that the density of footprints at a particular location on mRNA is proportional to the time that ribosomes spend at this location. Therefore, it is possible to calculate the average density of ribosomes on specific codons to determine their relative decoding rates. All ribosome profiling studies that addressed this issue agree that there is little relationship between codon usage frequencies and their decoding rates.^10^,^23^,^60^,^61^ This is contrary to the widespread belief that rare codons should be decoded slowly, which most likely originated from the notion that highly expressed genes have more pronounced codon usage bias.^62^ However, the lack of correlation between codon frequencies and efficiencies is not so surprising. Very early studies of translation speed and accuracy have shown that it is the availability of cognate tRNAs, rather than the frequency of codons that modulates the rate of codon decoding.^63^ Jon Gallant introduced the term ‘hungry codon’ to discriminate between the two types of codons.^64^ Several computational studies employed the data obtained with ribosome profiling to explore the relationship between codon frequencies, availability of cognate tRNAs and decoding and translation rates.^59^,^65^,^66^ Stadler and Fire^61^ carried out ribosome profiling in *C. elegans* in order to provide evidence in support of the hypothesis that translation is slowed down by wobble interactions between a codon and its anticodon.^61^ A discussion of ribosome profiling data in relation to codon usage can be found in a recent comprehensive review by Plotkin and Kudla.^67^

**FIGURE 6:**
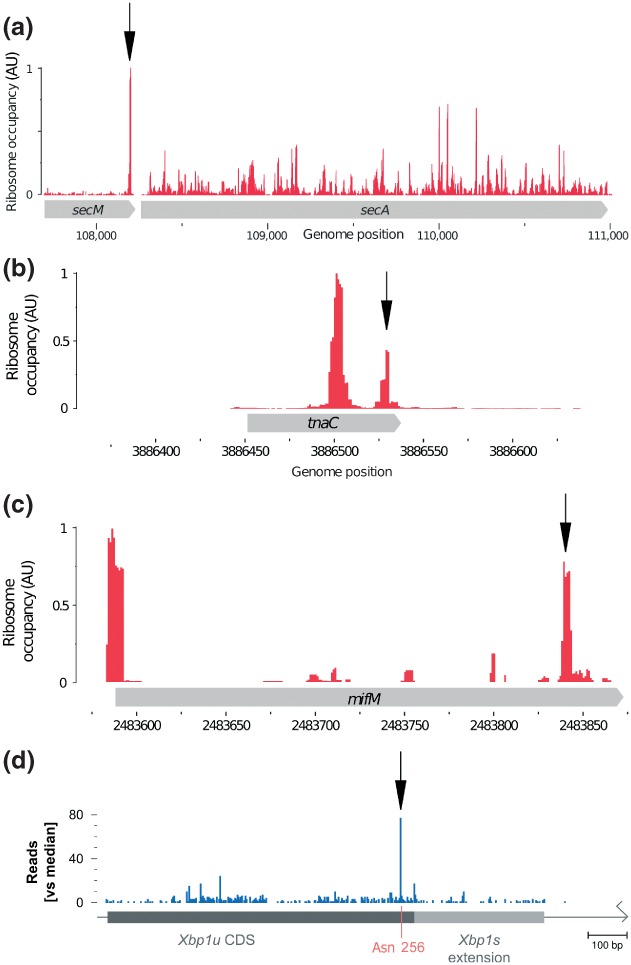
The increased ribosome density at known sites of ribosome stalling: *secM* (a) and *tnaC* (b) in *E. coli*; *mifM* (c) in *B. subtilis*; and *Xbp1* in *Mus musculus* (d). Black arrows indicate the locations of known ribosome pause sites (a–c: Reprinted with permission from Ref 60. Copyright 2012 Mcmillan Publishers Ltd; d: Reprinted with permission from Ref 10. Copyright 2011 Elsevier)

The truly unexpected observation generated by ribosome profiling was the realization that the rate of cognate tRNA selection in the A-site tRNA may not be the major factor that determines local translation elongation rates. Li et al.^60^ generated ribosome profiles in *Escherichia coli* and *Bacillus subtilis* and found that the ribosome occupancy at mRNA locations correlate with purine rich Shine–Dalgarno (SD) regions upstream of the A-site codons. The SD sequence is well known for its role in translation initiation in most prokaryotes^68^ and has previously been shown to affect elongating ribosomes.^69^ When it is located upstream of initiation codons it serves for anchoring initiating ribosomes by interacting with the complementary anti-Shine–Dalgarno (aSD) sequence in 16S rRNA. By performing a set of experiments, including ribosome profiling carried out for mRNA translated with orthogonal ribosomes (containing an altered aSD sequence), Li et al. have been able to demonstrate that SD sites indeed slow down elongating ribosomes. Under conditions of fast bacterial growth, the SD effect greatly exceeds that of particular codons.^60^ Ingolia et al.^10^ also have been able to identify a number of ribosome pausing sites using ribosome profiles from mESCs. Although the pause sites are enriched for glutamate and aspartate codons in the A site, enrichment for particular amino acids encoded by a sequence just upstream is yet another feature that is not directly related to the identity of a codon in the A-site. Notably, both studies confirmed increased ribosome density at known sites of ribosome stalling. Figure 6 shows the peptide-mediated stalling at *secM*^70^ and *tnaC*^71^ in *E. coli*, at *mifM* in *B. subtilis*^72^ and at *Xbp1* mRNA^73^ in mESCs, thus confirming the applicability of ribosome profiling for the identification of ribosome pausing sites.

All studies where ribosome profiling is used for estimating local decoding rates require the detection of the A-site codon location. Ribosome profiling does not provide direct information on the locations of the A-site codons. It is inferred from the locations of ribosome footprints. At present there are two strategies. One, used in ribosome profiling in eukaryotes, sets an offset between the 5′-end of the ribosome footprint and the expected location of the A-site codon. The offset is derived from the distance between the major density peaks for the 5′-ends upstream of the starts of main coding regions (in some studies stratified according to RPF length), see Refs 1,61 for details. The other, the so-called centre-weighted approach, was used for ribosome profiling in bacteria. In this case, the centre of the ribosome footprint is considered as the most probable location of the A-site, with codons adjacent to the centre also taken into account as potential A-site codons but with reduced weighting co-efficient, see Ref 60 for details. Recently, it has been found that in bacteria, Shine–Dalgarno sequences could affect the size and symmetry of ribosome footprints,^74^ thus potentially affecting the positions of the A-sites relative to the footprint ends. To what extent this phenomenon affects the above mentioned methods of A-site codon position detection needs further investigation.

### Selective Ribosome Profiling

Oh et al.^11^ introduced a procedure that they termed ‘selective ribosome profiling’. To obtain information on ribosome-associated chaperone trigger factor (TF) targets, Oh et al.^11^ combined ribosome profiling with affinity purification of the ribosomes bound with TF, thus mapping the locations of TF bound ribosomes on *E. coli* mRNAs. They found that in the majority of mRNAs, TF binds to the nascent peptide chain after the ribosome finishes translating about a hundred codons. TF was also found to have a strong preference for binding to ribosomes translating outer-membrane protein mRNA. To study cotranslational protein folding in mammalian cells, Han et al.^55^ developed the folding-associated cotranslational sequencing (FactSeq) technique. In this technique a specific folding is used as an affinity tag for isolating ribosomes along with protected mRNA fragments. Han et al.^55^ were able to use this technique to monitor the folding of hemagglutinin along its mRNA. Using a similar concept, Reid and Nicchitta^75^ carried out ribosome profiling after separating endoplasmic reticulum (ER) and cytosolic polysome fractions. Consequently, Reid and Nicchitta^75^ were able to identify the contribution of the two cellular compartments to global protein synthesis and found that preferential translation occurs on ER-bound ribosomes. Many mRNAs encoding cytosolic proteins are loaded with ribosomes on the ER and while mRNA abundance is higher in the cytosol, the ER-localized mRNAs have a higher ribosome density. Based on their findings, Reid and Nicchitta^75^ proposed that the partitioning of mRNAs between the cytosol and ER compartments is a mechanism of post-transcriptional regulation of gene expression: while protein synthesis preferentially occurs in the ER, mRNA storage and degradation occur in the cytosol.

These three studies have demonstrated the applicability of selective ribosome profiling for studying the compartmentalization of translation inside the cell as well as for elucidating the functional properties of ribosome associated factors.

### Identification of Novel Translated ORFs

The analysis of ribosome profiling data does not necessarily depend on gene annotation and thus can be used for the verification of existing gene annotations and the identification of novel nonannotated genome features such as protein-coding genes or short translated ORFs. *Ab initio* annotation of genomes is particularly difficult for short ORFs because short ORFs could exist purely by chance and information on the nucleotide composition of short ORFs may not be sufficient to discriminate coding from noncoding ORFs. Ribosome profiling provides a way to find translated ORFs irrespective of their length. Most recoded genes that require nonstandard translational events, such as programmed ribosomal frameshifting, cannot be automatically identified with pure sequence analysis because of the high diversity and our poor understanding of recoding signals. Ribo-seq can be used to facilitate the discovery of novel recoded genes. It has been argued that most alternative splice isoforms may not contribute to protein synthesis.^76^ Identifying those that are productive is not trivial. In the following sections we discuss how ribosome profiling can provide data that can be used to discriminate translated isoforms from those that are untranslated. In addition, we review how ribosome profiling data can be used to explore the evolution of protein coding genes.

#### uORFs, nORFs and Novel Protein Coding Genes

Protein coding genes are usually discriminated from regulatory ORFs. While it is becoming increasingly difficult to reach agreement on a formal definition of a gene,^77^ it is colloquially used as a term for a sequence that encodes a functional protein molecule. Thus, a regulatory ORF is distinct in the sense that its translation (rather than the product of that translation) is functionally important. Clearly, the distinction is not strict. In prokaryotes, where polycistronic mRNAs are abundant, the translation of adjacent ORFs encoding functional protein products is often coupled providing a regulatory mechanism for their co-expression. It is also possible that the translation of some short regulatory ORFs in eukaryotes may result in the biosynthesis of biologically active peptides. Ribosome profiling alone does not provide information regarding the function or importance of the translated ORF product. The distinction needs to be made based on other factors such as the organization of adjacent ORFs, phylogenetic conservation, etc. Therefore we describe the detection of regulatory ORFs and novel protein coding genes in the same section.

**FIGURE 7:**
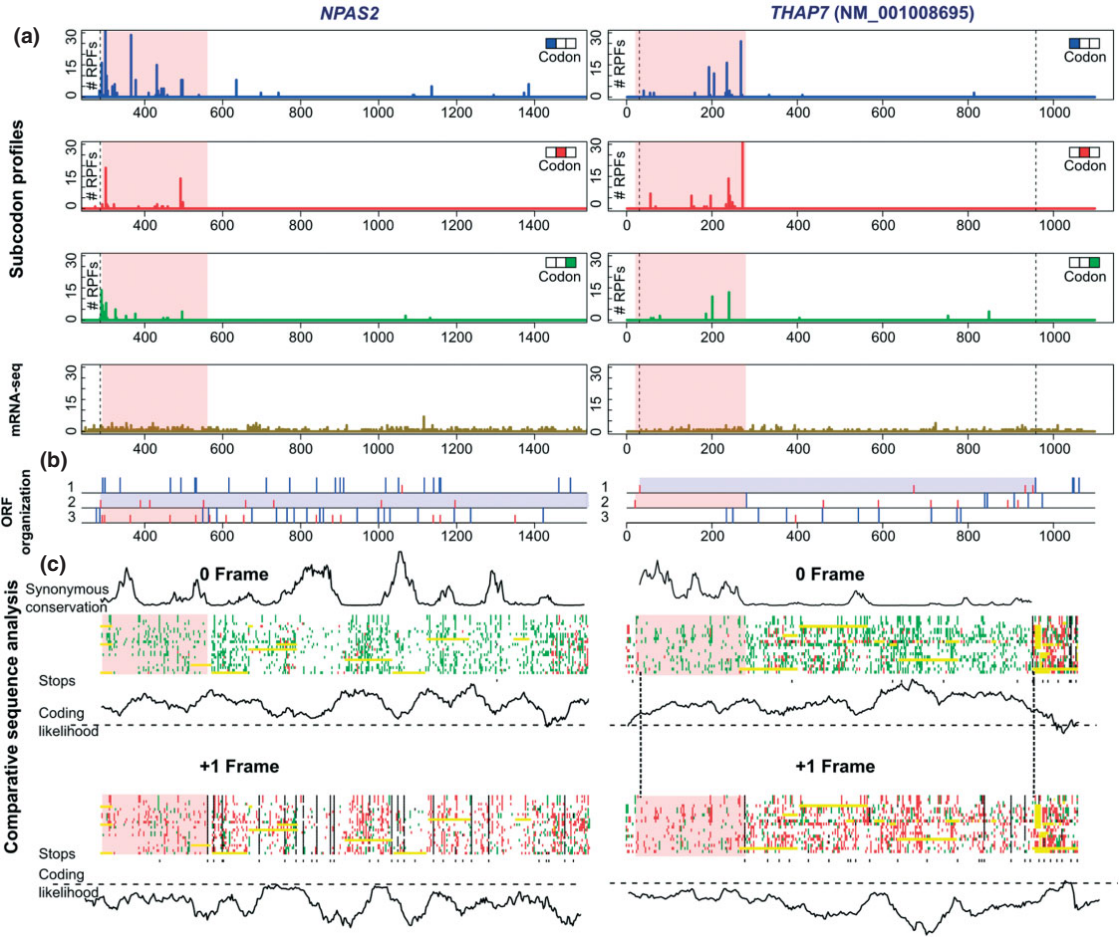
Subcodon ribosome profiles for human *NPAS2* (left-hand side) and *THAP7* (right-hand side) mRNAs. The triplet periodicity of ribosome profiles allows the discrimination of the translated reading frame by separating footprints into subcodon positions depending on the phase of their 5′-ends (a). In both cases, the subcodon profiles exhibit the pattern consistent with translation of alternative ORFs (highlighted in pink in b). The functionality of these two ORFs is supported by deep phylogenetic conservation that is evident from the comparative sequence alignments shown in (c) (Reprinted with permission from Ref 52. Copyright 2012 Cold Spring Harbor Laboratory Press)

The very first ribosome profiling study in yeast^1^ revealed the occurrence of extensive translational events in the 5′ leaders of eukaryotic mRNAs that was confirmed by all subsequent eukaryotic ribo-seq datasets. These translational events appeared to be very sensitive to changes in environmental conditions suggesting a regulatory role of the 5′ translation.^1^,^10^,^23^,^48^ While the current ribosome profiling studies point to the existence of a large number of translated short uORFs, their identification appears to be difficult. uORF's short length, limited footprint coverage, frequent non-AUG initiation, and the simultaneous translation of overlapping ORFs are among the many factors complicating the unambiguous assignment of ribosome footprints to one of several potential translated uORFs. In principle, the triplet periodicity of ribosome footprints allows the detection of the translated reading frame and this feature could help in the identification of short translated ORFs. Michel et al.^52^ have demonstrated that given sufficient coverage, it is possible to use triplet periodicity for detecting the translation of reading frames alternative to the main one. The ability to predict alternatively translated frames depends on sufficient coverage, length of ORFs overlap and the relative intensity of the alternative frame translation. Despite these limitations, Michel et al. not only detected several uORFs translated at an efficiency higher than the main protein product ORF, but also ORFs with initiation codons downstream of the main ORF start codon which they termed nORFs (for nonupstream regulatory ORFs) (see Figure 7). It is as yet unclear how such nORFs could regulate the translation of main ORFs although their functional importance is supported by phylogenetic analysis. Comparative analysis of one such nORF in *NPAS2*, a gene encoding a component of the suprachiasmatic circadian clock in mammals, provides evidence for the conservation of the nORF rather than its protein sequence suggesting a role for its translation, but not for its product,^52^ see Figure 7c.

Because splicing in bacteria is uncommon, sequences of bacterial ribosome footprints can be aligned directly to genomic sequences, thus simplifying the discovery of novel protein coding genes. Strikingly the first ribosome profiling study performed in *E. coli*^11^ revealed several protein coding genes that were not annotated previously despite *E. coli* K12 being one of the most extensively studied organisms with an intensively annotated genome. Hence it is evident that current sequence analyses approaches do not allow the identification of all protein coding genes based on DNA sequences even in a well-studied bacterial species and that ribosome profiling is capable of improving the situation. This was further exemplified with a recent study of human cytomegalovirus (HCMV) infection where ribosome profiling of elongating and initiating ribosomes increased the number of identified translated ORFs by more than a third.^14^

#### Correcting Annotations of Existing Genes and Detecting Protein Isoforms

Ribosome profiling of elongating ribosomes has significant limitations for the analysis of initiation codons. When protein synthesis is initiated from multiple start codons, only the 5′-end start codon can be identified. Therefore, ribosome profiling of initiating ribosomes (described in the section *Ribosome Profiling of Initiating Ribosomes* of this review) is much more appropriate for this goal.

**FIGURE 8:**
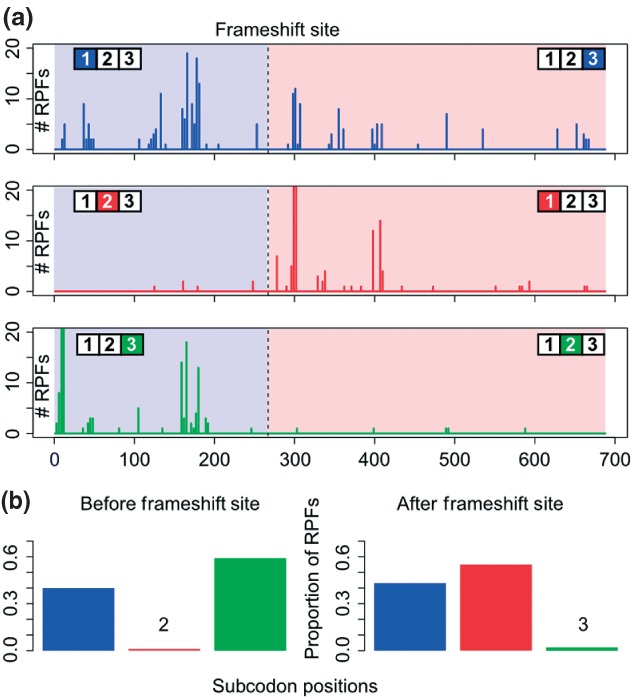
The utilization of triplet periodicity for detecting transitions in translated reading frames. Panel (a) shows the absolute number of RPFs aligning to each subcodon position for the coding region of human antizyme 1 (*OAZ1*) mRNA. The location of the programmed ribosomal frameshift site is indicated by a broken black line. Panel (b) shows the distribution of the number of RPFs aligning to different sub-codon positions, upstream of the frameshift site (left) and downstream (right). It can be seen that the sub-codon position with the lowest RPF count shifts from the second to the third upon ribosomal frameshifting which is consistent with the +1 directionality of the programmed ribosomal frameshift utilized by *OAZ1* in its expression (Reprinted with permission from Ref 52. Copyright 2012 Cold Spring Harbor Laboratory Press)

In contrast to determining the 5′ boundary of a protein coding region, ribosome profiling of initiating ribosomes provides no value for finding the 3′ boundaries of coding regions. Identifying the 3′ boundary of coding regions is problematic in the case of recoding events (see Ref 78 for a compilation of reviews on Recoding). The meaning of stop codons is known to be redefined with the recoding *cis*-elements to either standard (stop codon readthrough) or to nonstandard proteinogenic amino acids (selenocysteine and pyrrolysine insertions). In addition, in the case of programmed ribosomal frameshifting, a portion of the ribosomes shift frames at specific locations in the mRNA thus terminating at a stop codon that is out-of-frame relative to the initiator codon. Michel et al.^52^ developed a method for identifying frame transitions in mRNA translation based on the triplet periodicity of ribosome profiling and demonstrated its applicability by finding known cases of ribosomal frameshifting in humans (see Figure 8) as well as a set of human mRNAs with translated overlapping ORFs. Using a similar approach, Gerashchenko et al.^23^ identified four novel cases of ribosomal frameshifting in yeast (*APE2*, *MMT2*, *URA8*, and *YLR179C*). Moreover, the identified cases appear to be dependent on oxidative stress suggesting that ribosomal frameshifting plays a regulatory role in these recoded genes.^23^

As suggested by Ingolia et al.,^1^ the marked absence of RPFs in unspliced introns helps discriminate between alternative splice forms. When multiple isoforms exist for a given gene, ribosome profiling in conjunction with mRNA-seq, can help in the correct identification of the transcribed and translated isoform. Ribosome profiling can also be useful for discovering novel translated mRNA variants. By analyzing the triplet periodicity in the ribosome profile of the human gene *C11orf48*, Michel et al. found that 3′-terminal exons are predominantly translated in a frame that is alternative to the predicted. More detailed analysis of available transcripts revealed the existence of an mRNA variant with an additional exon due to an alternative transcription initiation site. This shorter variant is translated in an alternative frame, resulting in dual decoding of the last three exons of *C11orf48*. The peptide generated from this additional exon has been independently detected with mass spectrometry.^79^

#### Non-mRNA Translation

Several studies have found RPFs aligning to genomic sequences that are not annotated as protein coding. Moreover, many are believed to be noncoding transcripts. This raises questions about the nature of this phenomenon, whether it reflects genuine translation and if it does, what is the function of such translation. A high proportion of the yeast noncoding genome is transcribed and these transcripts are termed stable unannotated transcripts, SUTs.^80^ Wilson and Masel^81^ have found that over half of all SUTs are associated with ribosomes, especially at AUG codons and proposed that this type of low level nondeleterious translation may facilitate *de novo* gene birth.

Carvunis et al. extended this idea further by proposing an evolutionary model of functional genes evolving *de novo* through transitory proto-genes.^82^ Signatures of translation have been found for 1,139 of total ∼108,000 unannotated ORFs (>10 codons) in *S. cerevisiae* outside of annotated features on the same strand. To find evidence for proto-gene mediated evolution, Carvunis et al. estimated the order of ORF emergence in *S. cerevisiae* using their conservation among Ascomycota.^82^

Evidence of translation in presumed noncoding regions in mammals has also been found. Ingolia et al.^10^ observed RPFs on >1000 large intergenic noncoding RNAs (lincRNAs) in mESCs and proposed to call them sprcRNAs for short polycistronic ribosome-associated coding RNAs to discriminate them from lincRNAs. Lee et al.^12^ also found evidence of ribosome association with presumed nonprotein-coding RNAs (ncRNAs) in HEK293 cells.

## RIBOSOME PROFILING OF INITIATING RIBOSOMES

Although to date there have been only four published works where ribosome profiling was carried out on initiating ribosomes, we dedicate this separate section of our review to the topic. As illustrated in Figure 3, this type of ribosome profiling provides information on mRNA translation that cannot be captured by the profiling of elongating ribosomes. Thus we believe that such experiments will be used as frequently as the original method, and more likely used in parallel. In terms of differential gene expression, initiation is slow in comparison with elongation (unless we consider special cases like ribosome pausing) and therefore is a rate limiting step. Thus, provided that it is accurately measured, the rate of initiation of translation in most cases would be a better predictor of translation rates than the density of elongating ribosomes on mRNAs. In terms of the characterization of protein products, it is also advantageous because the data on the locations of initiation codons can be easily interpreted to predict protein isoforms translated from different start codons. The main disadvantage of this method is its inability to provide direct information on local translation elongation rates and recoding events. Its utility for discriminating the translation of alternative splice variants is also limited.

The critical aspect of this strategy is a method for freezing initiating ribosomes. Several approaches have been used in eukaryotic systems. As yet, there have been no similar studies reported for bacteria.

### Mapping Translation Initiation Sites (TISs)

The first attempt to obtain a map of TISs using a direct experimental approach was made in mESCs with the drug harringtonine.^10^ Harringtonine binds to a 60S subunit and forms an 80s ribosomal complex with the initiator tRNA but blocks aminoacyl-tRNA binding in the A-site and peptide formation.^83^ To identify translation initiation codons precisely, Ingolia et al. used a support vector machine (SVM) learning technique and reported 13,454 unique TISs within ∼5000 well-expressed transcripts. The majority (65%) of these transcripts contain more than one detectable TIS with 16% containing four or more sites. Extensive translation initiation at non-AUG codons was also observed, particularly upstream of annotated starts. A potential problem with this approach is that because harringtonine binds to the 60S subunit, its binding could affect the selection of initiation codons by the ribosome.

To avoid any potential selection effect of harringtonine on initiation codons, Fritsch et al.^13^ mapped TISs by enriching elongating ribosomes near start codons instead of blocking initiating ribosomes. For this purpose puromycin was used to induce premature termination of elongating ribosomes which resulted in a relative increase in ribosome density at a few codons downstream of the TISs. These ribosomes were blocked with cycloheximide prior to nuclease treatment. The identification of TISs was carried out with a machine learning technique based on neural networks yielding 7471 unique TISs in 5062 well-expressed transcripts in a human monocytic cell line. Only 30% of non-CDS-overlapping uORFs initiated with AUG and only 8% of CDS-overlapping uORFs initiated with AUG. This finding supports the earlier result^10^ regarding the abundance of non-AUG initiation in 5′ leaders.

To obtain TIS maps, Lee et al.^12^ used a different drug, lactimidomycin, which binds to 80S ribosomal subunits after its assembly on start codons, making any bias on the selection of start codons less likely in comparison with harringtonine. To improve the lactimidomycin TIS signal detection, initiating ribosome footprints were compared with elongating ribosome footprints generated with cycloheximide treatment carried out in parallel. From ∼10,000 transcripts with detectable TIS peaks, Lee et al. identified a total of 16,863 TISs.^12^

**FIGURE 9:**
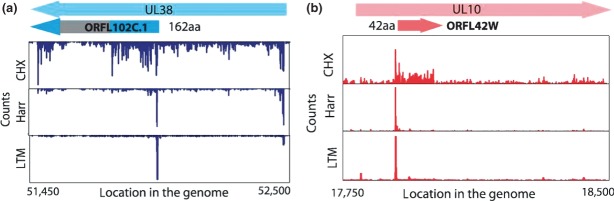
The ribosome initiating profiles [harringtonine (Harr) and lactimidomycin (LTM)] and elongating profiles [cycloheximide (CHX)] for the HCMV genes *UL38* (a) and *UL10* (b). The two ribosome profiling approaches aided the identification of internal initiation sites in both genes, with an N-terminally truncated translation product for *UL38* and a previously unknown out-of-frame translated ORF contained within the *UL10* gene (Reprinted with permission from Ref 14. Copyright 2012 AAAS.)

In experiments carried out in HCMV-infected cells, Stern-Ginossar et al. used both harringtonine and lactimidomycin treatments and found the results comparable: >98% of the initiation sites detected using harringtonine were also detected using lactimidomycin.^14^ So although the mechanism of action of the two drugs is different, they arrest ribosomes mostly at the same locations. Stern-Ginossar et al. also generated ribosome elongation profiles of mRNAs pretreated with either cycloheximide or lysed without drug pretreatment. Together their separate profiles of initiating ribosomes and elongating ribosomes enabled the identification of hundreds of previously unidentified ORFs in HCMV, including internal ORFs lying within existing ORFs (nORFs), short uORFs, ORFs within transcripts antisense to canonical ORFs and previously unidentified short ORFs encoded by distinct transcripts (see Figure 9).

#### uORFs, nORFs, and Novel Genes

As long as no recoding events are involved in the translation of an mRNA transcript (i.e., the triplet periodicity of translation is maintained and amino acids are not incorporated at stop codons), the identification of translated ORFs can be made based on TIS detection. Moreover it is even simpler in comparison with ribosome footprints obtained with elongating ribosomes. Because ORFs overlap, it is very difficult to discriminate between the translation of a single frame and the translation of two overlapping ORFs occupying the same transcript location. If TISs are detected with codon precision, information regarding the framing can be determined and therefore can be used for the identification of translated ORFs.

All of the studies in the previous subsection reported the existence of ORFs in different configurations relative to the main annotated ORFs with the largest proportion of them being uORFs.^10^,^12^–^14^ However, novel ORFs located downstream have also been detected raising questions regarding their importance.^52^

In many cases translation initiates on very short ORFs, which are unlikely to produce functional peptides: among 751 translated ORFs in cytomegalovirus, 245 are shorter than 21 codons, 239 are in the range of 21–80 and only 120 are longer than 80 codons.^14^ The translation of many of these ORFs may represent gene expression noise and the products of these ORFs may have no function. They could, however, be potential targets for the host immune response and are of interest for understanding the biology of the virus.

#### Non-AUG Translation

While initiation at non-AUG codons is frequent in many bacteria, as recently as 2010, the number of non-AUG codons identified as potential translation initiation sites in humans was small. In 2011, Ivanov et al.^84^ reported 42 novel non-AUG initiation sites which were detected with the analysis of evolutionary signatures of protein-coding sequences in the regions upstream of annotated codons. Ribosome profiling increased this number dramatically: the number of non-AUG TISs reported in the studies described here is close to a half of all TISs. In addition, non-AUG initiation occurs more frequently in uORFs. Lee et al. reported that over 74% of upstream TISs in human are non-AUG codons, often associated with short uORFs.^12^

#### Protein Isoforms

**FIGURE 10:**
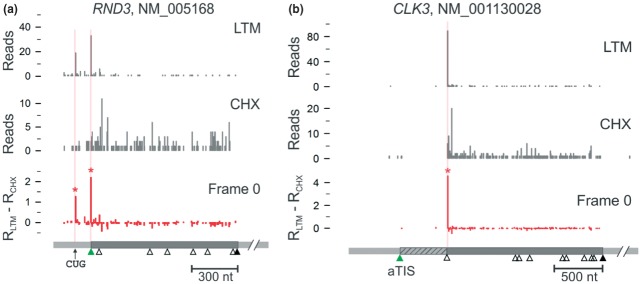
Detection of protein isoforms with alternative N-termini. Panel (a) shows an N-terminally extended isoform of the human *RND3* gene which has an in-frame CUG initiating codon. Panel (b) shows a truncated isoform of the human *CLK3* gene which was found to initiate at an AUG codon downstream of the annotated AUG start codon (Reprinted with permission from Ref 12. Copyright 2012 National Academy of Sciences USA.)

Figure 3 illustrates why ribosome profiling of initiating ribosomes is particularly suitable for the detection of alternative protein isoforms (extensions and truncations of annotated CDS). As discussed in the section *Ribosome Profiling of Initiating Ribosomes*, initiation at alternative sites both upstream and downstream of the annotated protein coding ORFs is pervasive. Many of these events were heretofore difficult to detect and annotate. Now, advancements can be made in gene annotations by incorporating ribosome profiling data. Figure 10a shows an N-terminally extended isoform of the human *RND3* gene which has an in-frame CUG initiating codon. Figure 10b shows a truncated isoform of the human *CLK3* gene which Lee et al.^12^ found to initiate at an AUG codon downstream of the annotated AUG start codon. Ingolia et al.^10^ identified 570 genes with potential N-terminal extensions and 870 with N-terminal truncations in the 4994 genes that were analyzed in mESCs. Fritsch et al.^13^ also reported 546 N-terminal protein extensions in human (regions downstream of annotated starts were not analyzed). These examples highlight the usefulness of ribosome profiling data in improving existing annotations.

## PERSPECTIVES

Translation is a complex process and therefore its characterization will require the use of a combination of approaches. Ribosome profiling of elongating and initiating ribosomes was carried out in parallel in the most recent study.^14^ The combination of the two approaches benefits from the specific advantages of each method. Moreover, it is very likely that further variants of ribosome profiling will be developed in order to capture the characteristics of translation that are unattainable by the methods described in this review.

Translation is a process that is downstream of transcription and therefore it cannot be characterized accurately without information on the transcriptome. Therefore transcriptome sequencing and ribosomal profiling have to be carried out in parallel. Combined together, RNA-seq and different ribo-seq techniques will form a universal set of tools for characterizing the molecular state of any living cell at a very detailed level. The continual reduction in cost and time of nucleic acid sequencing will ensure the accessibility of these techniques for gene expression measurements to a very wide research community. There is little doubt that the application of this suite of techniques will grow explosively. However, the ease of the data generation will demand adequate capacity to process, interpret, store, integrate and distribute the data.^85^
